# Assessing newly introduced climate change adaptation strategy packages among rural households: Evidence from Kaou local government area, Tahoua State, Niger Republic

**DOI:** 10.4102/jamba.v9i1.383

**Published:** 2017-04-25

**Authors:** Ali M. Tabbo, Zakou Amadou

**Affiliations:** 1 Department of Rural Sociology and Economics, Abdou Moumouni University, Niger; 2 Department of Rural Sociology and Economics, Tahoua University, Niger

## Abstract

This research discusses new strategies developed and introduced by national and international partners to help farmers in building adaptative capacity against the negative externalities of climate change. The purpose of this study is to determine and to assess the most important adaptation strategies introduced by development partners. Based on the recognition interview with farmers and synthesis of previous research, 13 strategies were compiled and included in the study. Thus, an advance in the balanced incomplete block design was used to design the questionnaire served as data collection tools. For each question, respondents were asked to choose their best and their worst strategies. Thus, the difference between the best and worst strategies consistent with random utility theory was used for the modelling. Results show that herd rebuilding, human capacity building, introduction of fishing, water and soil conservation activities, introduction of leafy vegetable such as *Moringa oleifera*, financial credit, forage seed marketing and introduction of agriculture inputs were the most important strategies, while the support to vegetable production, income-generating activities, the use of agricultural improved seed varieties, anti-fire band making and cereal bank introduction were the least important adaptation strategies for farmers. These results are therefore essential for the dissemination of adaptation strategies, thereby stimulating and maintaining sustainability development actions in the study area.

## Introduction and background

Several studies have reported that climate change is a global externality that negatively affects households, communities and larger economies and that its potential to disorganise economies and public finances is real and that its challenge at the global, the national and the local levels can no longer be denied (Elasha et al. 2006; Sultan & Gaetani 2016). This challenge is so huge that concerted efforts have been undertaken to reduce its negative externalities on vulnerable households through designing and disseminating adaptation strategies.

At the global level, a decision to discuss climate change was reached in November 2006 at the United Nations Framework Convention on Climate Change held in Nairobi, where the issue of climate change was brought to the attention of the international community. It is widely recognised that developing countries will suffer disproportionately from the effects of climate change as compared to their counterparts. These developing countries are not only in a weak position to reduce the negative effects, but they will also lose some gains generated by the current development. Furthermore, the Paris deal has recently been adopted, and the final draft includes a temperature limit ‘well below 2 degree Celsius’, an imposition of legal obligation on developed countries to provide climate finance to developing countries, a mitigation through binding parties to prepare and regularly update climate engagement, a global goal on adaptation via reinforcing adaptive capacity and strengthening resilience and reducing vulnerability to climate change (International Summit on Climate Change held in Paris 2015).

At the national level, the Niger government has started to show a strong leadership on environmental issues related to the adverse effects of climate change even before the Nairobi Summit. Thus, the first meeting held in Maradi in 1984^1^ was aimed at shedding more light on the short-term and long-term consequences of climate change and developing adaptation strategies. After two decades, with the support of development partners such as United Nation Development Program (UNDP), World Food Program (WFP), Food and Agricultural Organization (FAO), European Union (EU), United States Agency for International Development (USAID), Canada International Development Agency (CIDA), International Fund for Agricultural Development (IFAD), New Partnership for African Development (NEPAD) projects and programs, Community Action Programs (CAP) phases I, II and III as well as non-governmental organisations (NGOs), several climate change adaptation technologies have been designed, disseminated and tested. The Niger government has also introduced a new policy aimed at developing and reforming production systems in the rural sector. The experiences gained from this study have qualified the study area to be considered as a laboratory where the resilience of rural households as well as agricultural production systems becomes a reality.

At the local level, the new vision of development focuses on a better understanding of the people’s aspirations and their direct involvement in the development process. It is argued that people make the project and the project ensures the development of the land. Kandlinkar and Risbey (2000) stated that adaptation strategies assist farmers to achieve their food, income and livelihood security objectives and thereby maintain these livelihoods in the face of changing climatic and socio-economic conditions. Farmers can also reduce the potential damage by making tactical responses to these changes. Additionally, Brockmann,^2^ president of the United Nations General Assembly to the Indigenous Peoples’ Global Summit on Climate Change in 2009, said that climate change poses threats and dangers to the survival of indigenous communities worldwide, even though they contribute the least to greenhouse emissions.

A large body of research has also been devoted to climate change adaptation and mitigation strategies. Thus, a study by Lethoko (2016) revealed that climate change adaptation and mitigation strategies have not been included in these surveyed municipalities in South Africa though being the most vulnerable in the study area. Aleke and Nhamo (2016) have also studied information and communication technology (ICT) and climate change. They concluded that ICT intervention must play a key role in the ongoing climate change strategies in the South African mining sector. In addition, Angula and Kaundjua (2016) have examined factors contributing to subsistence farmers’ vulnerability to climate change impacts. They concluded that households interviewed showed low level of adaptive capacity as a result of combined effects of cultural, social, economic and political factors as well as constant exposure to climate change risks. Ncube et al. (2016) also investigated climate change, household vulnerability and smart agriculture in South Africa. They first argued that the impact of climate change at the local level is not well-documented and that rural household vulnerability to climate change is not well-understood. Their findings revealed that rural household without capital is the most vulnerable and that their movement towards smart agriculture should be encouraged as the most sustainable adaptation and mitigation strategies. Matsvange et al. (2016) also studied the impact of forests on communities from Nyanga, Guruve and Zvimba districts of Zimbabwe. Results from their study indicated that actions such communities’ initiatives have been undertaken to halt the high rate of deforestation and thereby to protect and to sustain forest and land resources from which they derive economic and social benefits. Watson et al. (1998) also have assessed the effect of climate change on in water availability, crop yields and inundation of coastal areas. They have also argued that all these areas will have further indirect effects on food security and human health. They also concluded that adaptive options including new temperature, pest-resistant, crop varieties, and new technologies to reduce crop yield as well as integrated approaches have been proposed for current and longer-tern management to river basin and coastal zone. Finally, Sall et al. (2011) investigated how the impact of climate change exacerbates existing vulnerabilities and how adaptation strategies thought at global level and implemented at local level are necessary. They have highlighted that adaptation strategies should consider interrelations and local differences by assessing not only socio-economic mutations, but also environmental mutations induced by this situation on the lives of people and make sure that the sustainability of adaptation strategies and capacities of various actors being implemented.

Analysing adaptation strategies is therefore an important way to help rural farmers reinforce their resilience capacity. However, the replicability and sustainability of adaptation packages depend not only on the cultural context in which they are envisaged to be disseminated but also on the direct involvement of farmers. This is why farmer’s views are capital for projects’ success, and this study will help to correct any deficiency observed in project planning and implementing in the study area. This current study will also contribute to compensate and reinforce weaknesses observed in the implementation of local adaptation strategies and to subsequently determine combination of strategies which is more welfare enhancing for the rural household. The overall objective of this study is to determine and assess the most important adaptation and mitigation strategies disseminated by development partners to enhance vulnerable rural households’ resilience capacity against the negative impacts of climate change.

## Conceptual framework

We assume that farmers maximise their utility when they decide to choose one strategy as the best and another as the worst to reinforce their resilience capacity against the negative effect of climate change. Therefore, NGOs, the government and development partners should assess what strategies are the most important to farmers before developing and releasing adaptation packages. This will also assist them to efficiently and effectively channel scare resources and avoid wasting time and effort in implementing adaptation strategies yielding no positive impact on farmers’ welfare. Furthermore, the understanding and the determination of the most important adaptation strategies will help farmers to adopt economically feasible and environment-friendly projects capable of stimulating development process in the study area and beyond. The application of best and worst scaling has been widely utilised to study consumers’ behaviour because it is consistent with consumer theory and random utility theory. Based on this approach, a set of adaptation strategies is presented to farmers, and they are asked to choose their best and worst strategies (Lusk & Bridgeman 2009). The method of the best and worst strategy has recently been utilised to determine the relative importance of food values to consumers (Lusk & Bridgeman 2009) and farmers’ preferences about their own strategies against negative effects of climate change (Tabbo, Amadou & Danbaky 2016).

Therefore, farmers are maximising utility when choosing their best and worst adaptation strategies in a set of strategies observed in each question. Thus, the choice made by each farmer can be mathematically expressed:

$$U_{ij}=V_{ij}+\varepsilon_{ij}$$ [Eqn 1]

where *U_ij_* is utility of farmer in choosing a given strategy j, *V_ij_*and ε_*ij*_ are, respectively, the deterministic and stochastic components and of utility.

## Data collection method

The method of best and worst scaling allowed farmers to better cope in determining the best and worst strategies been utilised by farmers to cope better against the negative effects of climate change. Thus, the balanced incomplete block design was used to design the questionnaire which served as a tool for data collection. This block design is balanced with respect to rows, and each strategy is equally replicated through the questionnaire thereby maintaining the likelihood principle. Based on previous studies and interviews with farmers and development partners, 13 have been compiled and used in this study. The R statistical software (R is a free software environment for statistical computing and graphics) was used to generate the 13 blocks or questions having four strategies randomly assigned. For each question, farmers are asked to choose their best and worst adaptation strategies.

**FIGURE 1:**
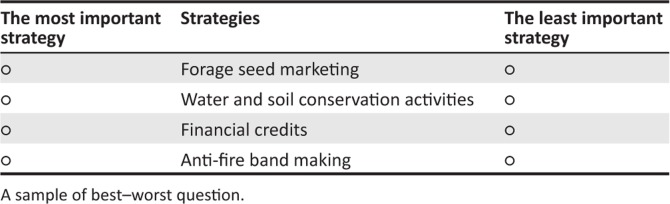
Please choose your best and worst strategies to adapt to climate change.

The survey was conducted in two villages, Kao and Ichanchar, located in the municipality of Kaou. The county of Kaou was selected because it is the most vulnerable municipality in Tahoua State. Respondents were randomly selected to participate in this survey. To increase the diversity of our sample, a specific gender within a given household was targeted. This approach is important because it gives the opportunity for rural women to express their opinions in a world, which is largely dominated by the views of men. In total, 100 farmers were randomly selected and interviewed face-to-face. A sample based on best and worst scaling is presented in Figure 1.

## Econometric methods

Mixed multinomial logit or random parameter model was used to analyse our choice experiment because it is capable of relaxing the independently identically distributed (iid) assumption and approximating any random utility model to any degree of accuracy (McFadden & Train 2000).

By following Lusk and Briggeman (2009), in a set of *k* elements, there are *k*(*k* − 1) possible best–worst combinations. The choice of a pair of strategies in the *k*(*k* − 1) corresponds to a maximum allocation of the choice difference. We assume that *α_k_* represents the location of value *k* on a specific scale of importance. Thus, the level of importance for each individual person *i* can be expressed as follows:

$$P_{ik}=\alpha_{k}+\varepsilon_{ik}$$ [Eqn 2]

where ε_*ik*_ is a random term included to take into account unobserved factors.

The probability that *k* and *j* be selected in a set as best and worst is equal to the probability that the difference between *P_ik_* and *P_ij_* must be greater to all other *k*(*k* − 1) − 1 options in the choice set. Assuming that the error term has an iid distribution, a multinomial logit model can be used in the probability expressed as follows:

$$\text{Prob }(k\text{ is chosed best and }j\text{ worst})=\frac{\exp(\beta_{k}-\beta_{j})}{\sum_{i=1}^{k}\sum_{m=1}^{k}(\beta_{l}-\beta_{m})-k}$$ [Eqn 3]

This regression analysis was used to determine the relationship among several pair variables and then identify how a change from one affects the other. The estimation of the model helps to determine which attribute is the most preferred and which is the least preferred. Results from the estimated mixed logit model were used to rank preference because it is capable of approximating any behaviour model and relaxing the independence irrelevant alternative assumption. Thus, preference share was calculated based on the following:

$$\text{Prob }(\text{Strategy }j\text{ is chcosen})=\frac{e^{X\beta_{j}}}{\sum_{k=1}^{J}e^{X\beta_{k}}}$$ [Eqn 4]

where *V_j_* = *X*β_*j*_ is the utility of the strategy *j* and *V_k_* = *X*β_*k*_ is the utility of the strategy *k*.

Finally, the standard normal distribution has been used to determine the percentage of farmers having positive and negative preference for a given strategy. Thus, the ratio between the mean and standard deviation (following the normal distribution) for each strategy has served to determine the probabilities above and below zero.

## Results and discussions

This section presents summary statistics of surveyed respondents, the mixed logit model, multinomial logit model, the probability distribution for each strategy and heteroscedastic extreme value (HEV) model. Table 1 provides summary statistics of key variables. Results show that most of the respondents were male (71.8%), were married (85.4%) and were uneducated (94.6%). They had an average age of 46 years and with an average monthly income of 63 000 Franc of the French colonies of Africa (FFCA)

**TABLE 1:** Summary statistics of surveyed respondents.

Variables	Definition	Mean	Standard deviation
Age	Age in years	45.885	17.648
Gender	1 if male, 0 if female	0.719	0.452
Marital status	1 if married, 0 otherwise	0.854	0.355
Education	1 if educated, 0 otherwise	0.156	0.365
Income	Monthly income in 1000	63 000 FCFA	24.300

Note that $1 = 500FCFA.

Ninety-nine instead of hundred respondents were reported because one questionnaire was not usable.

*N* = 99.

Table 2 reports both mixed logit and multinomial models while Table 3 presents a heteroscedastic model. Results from likelihood ratio tests show that the mixed logit model outperformed both the multinomial and heteroscedastic models and, therefore, the only estimate from the mixed logit model as shown in Table 3 was retained for interpretation. As indicated in Table 2, coefficients with positive coefficients are preferred, while coefficients with negative signs are discarded. Results show that herd rebuilding (14.55%) followed by human empowerment (13.48%), introduction of fishing (10.62%), water and soil conservation activities (10.44%), introduction of leafy vegetable such as *Moringa oleifera* (10.02%) and financial credits (9.92%) are the most preferred strategies. However, support to vegetable production (4.40%), income-generating activities (3.40%), and the introduction of improved seed varieties anti-fire band making (2.67%) and cereal bank introduction, (1.95%) are the least preferred strategies. Table 2 also reports the importance or weight of each strategy relative to introduction of agricultural inputs. Results indicate that herd rebuilding is 15 times more important relative to the introduction of agricultural inputs for farmers, followed by human empowerment (14 times), introduction of fishing (11 times), water and soil conservation (11 times), introduction of Moringa (10 times) and financial credits (10 times). The support to vegetable production, income-generating activities, introduction of improved early maturing varieties, anti-fire band making and cereal bank introduction have all lower preference shares relative to the introduction of agricultural inputs.

**TABLE 2:** Importance of newly introduced climate change adaptation strategies for farmers based on mixed logit and multinomial logit estimates.

Strategies	Mixed logit model			Multinomial logit model		
	
	Estimates	SE	PS (%)	Estimates	SE	PS (%)
Herd rebuilding	0.826**	0.282	14.55	0.409**	0.096	11.27
Human capacity building	0.750**	0.191	13.48	0.391**	0.091	11.06
Introduction of fishing	0.512**	0.192	10.62	0.378**	0.094	10.93
Water and soil conservation activities	0.494**	0.182	10.44	0.000	-	9.29
Introduction of leafy vegetable such as *Moringa oleifera*	0.453*	0.324	10.02	0.216*	0.086	9.14
Financial credits	0.443**	0.158	9.92	0.200**	0.101	7.62
Forage seed marketing	0.364	0.200	9.16	0.018	0.096	7.51
Introduction of agricultural inputs	0.000	-	6.37	0.004	0.100	7.49
Support to vegetable production	−0.369	0.189	4.40	−0.182*	0.092	6.24
Income-generating activities	−0.629**	0.224	3.40	−0.238**	0.102	5.90
Introduction of improved seed varieties	−0.745**	0.267	3.02	−0.380**	0.091	5.12
Anti-fire band making	−0.871**	0.244	2.67	−0.555**	0.102	4.30
Cereal bank introduction	−1.182**	0.285	1.95	−0.593**	0.101	4.14
Numbers of individuals	99.000	-	-	99.000	-	-
Log-likelihood	−2944.000	-	-	−3054.000	-	-

*, ** , denote significant level at 5% and 1%, respectively. SE and PS stand for standard errors and preference shares respectively.

**TABLE 3:** The probability above and below the mean for each strategy.

Strategies	Estimates	s.d.	% above zero	% below zero
Herd rebuilding	0.826	0.640	0.90	0.10
Human capacity building	0.750	2.295	0.63	0.37
Introduction of fishing	0.512	3.078	0.57	0.43
Water and soil conservation activities	0.494	2.366	0.58	0.42
Introduction of *Moringa oleifera*	0.453	5.899	0.53	0.47
Financial credits	0.443	0.299	0.93	0.07
Forage seed marketing	0.364	0.533	0.75	0.25
Introduction of agricultural inputs	0.000	0.000	0.00	0.00
Support to vegetable production	−0.369	0.197	0.03	0.97
Income-generating activities	−0.629	0.381	0.05	0.95
Introduction of improved seed varieties	−0.745	3.497	0.42	0.58
Anti-fire band making	−0.871	0.877	0.16	0.84
Cereal bank introduction	−1.182	1.724	0.25	0.75

s.d., standard deviation.

The mean and standard deviation as shown in Table 3 were used to determine the preference share for each strategy. Results indicate that financial credits are preferred by 93% of farmers, against 90% for herd rebuilding, against 75% for forage seed marketing, against 63% for human capacity building, against 58% for water and soil conversation activities and against 57% for introduction of fishing. Conversely, the support to vegetable production as adaptation strategy is avoided by 97% of farmers, against 95% for income-generating activities, against 84% for anti-fire band making, against 75% for cereal bank introduction and against 58% for dissemination of improved seed varieties. These results revealed that financial credits, followed by herd rebuilding, forage seed marketing, human capacity building, water and soil conversation activities and the introduction of fishing are the most important adaptation strategies for farmers. In contrast, adaptation strategies such as support to vegetable production followed by income-generating activities, anti-fire band making, cereal bank introduction and dissemination of improved seed varieties were the least preferred adaptation strategies.

Table 4 reports the HEV model. Likelihood ratio test shows that the mixed logit model outperformed the HEV model. Therefore, estimates from the HEV model though being presented below are not interpreted. The scale parameters of each strategy were also reported. It is important to note that the error variance is proportional to the square of the scale parameter.

**TABLE 4:** Farmers’ utility for new climate change adaptation strategies based on the heteroscedastic (HEV) extreme estimates.

Strategies	Estimates	SE	Probability	PS (%)
Cereal bank introduction	−1.253	0.130	< 0.0001	3.59
Introduction of improved seed varieties	−1.238	0.141	< 0.0001	3.65
Forage seed marketing	−1.162	0.159	< 0.0001	3.94
Income-generating activities	−0.940	0.119	< 0.0001	4.91
Anti-fire band making	−0.846	0.111	< 0.0001	5.40
Introduction of agricultural inputs	−0.572	0.125	< 0.0001	7.11
Water and soil conservation activities	−0.480	0.115	< 0.0001	7.79
Introduction of fishing	−0.303	0.121	0.0119	9.29
Financial credits	−0.288	0.130	0.0270	9.44
Support to vegetable production	−0.274	0.107	0.0106	9.57
Herd rebuilding	−0.109	0.096	0.2577	11.29
Human capacity building	−0.096	0.110	0.3823	11.43
Introduction of *Moringa oleifera*	0.000	0.000	0.000	12.59
SCALE (forage seed marketing)	1.168	0.086	< 0.0001	-
SCALE (introduction of improved seed varieties)	0.800	0.055	< 0.0001	-
SCALE (introduction of agricultural inputs)	1.313	0.104	< 0.0001	-
SCALE (introduction of fishing)	1.188	0.082	< 0.0001	-
SCALE (water and soil conservation activities)	0.627	0.041	< 0.0001	-
SCALE (income-generating activities)	1.458	0.130	< 0.0001	-
SCALE (cereal bank introduction)	1.184	0.085	< 0.0001	-
SCALE (financial credits)	0.987	0.067	< 0.0001	-
SCALE (herd rebuilding)	1.684	0.224	< 0.0001	-
SCALE (human empowerment)	1.206	0.099	< 0.0001	-
SCALE (anti-fire band making)	1.555	0.121	< 0.0001	-
SCALE (support to vegetable production)	1.000	-	-	-
SCALE (introduction of *Moringa oleifera*)	1.000	-	-	-
Number of individuals	99.000	-	-	-
Log-likelihood	−2947.000	-	-	-

SE, standard errors; PS, preference shares.

## Summary and conclusion

Several adaptation strategies have been introduced by development partners, government and NGOs to assist vulnerable rural households to reinforce their resilience against the negative externalities of climate change. However, these adaptation strategy packages are often introduced without consulting farmers to whom they are intended to, resulting in duplication of the same strategy by several agencies leading to wastage of time and resources. The overall objective of this study is to determine and assess adaptation strategies developed and disseminated by development partners so as to better assist farmers to increase their resilience against negative consequences of climate change. Synthesis of previous research and focused group discussions with farmers and resourceful persons have helped to determine the most common adaptation strategies that were compiled and included in this study. The balanced incomplete block design approach, possessing four adaptation strategies randomly assigned to block or questions, was used as a data collection instrument. The difference between best–worst scaling setting, consistent with random utility theory, is used to analyse the data by fitting mixed logit model.

Results show that farmers are now aware that combining their local adaptation strategies and newly introduced adaptation strategies is important to build strong resilience against the negative externalities induced by climate change. Results reveal that herd rebuilding is the most important for farmers. This is illustrated by the increasing introduction of goats in the northern part of Niger where livestock such as goats have developed resilience because goats are easy to maintain, they are very prolific and they are capable of producing high-quality meat and milk. Goats are also known to impact environmental regeneration through their waste, which involves the return of the vegetation, and this idea is confirmed by research findings stating that the Sahel is getting more and more green. The second most important adaptation strategy for farmers is human capacity building. Farmers are aware that education is important to use technology gadgets for a better understanding of climate change process. Education and training also empower farmers with skills about how to read a pluviometer, to use agricultural inputs, to undertake irrigation and to conserve highly perishable agricultural products.

Results also reveal that farmers value the introduction of fishing as most important because it is not only beneficial to meet their nutritional food requirements, but also as a way to improve their source of income. Water and soil conservation activities are also valued as the most important strategy by farmers. This strategy is implemented through cash for asset or foods for asset, which keeps producing successful stories. Results also indicate that financial credits and forage seed marketing were also considered as most important adaptation strategies. Results finally showed that agricultural inputs are less valued by farmers because they prefer organic fertilisers obtained from animal wastes. Support for vegetable production as adaptation strategy is also considered as least preferred in the study area because access to water is still a challenge for the most rural household. This situation is also worsened by a shortage of rainfall observed in the village of Ichanchar where the introduction of improved seed varieties has resulted in limited success. Farmers also avoided anti-fire band making because it affects the quality of the forage. These findings should be considered to significantly increase farmers’ adaptation capacity in the study area.

The findings from this study, as well as local farmers’ adaptation strategies, will provide important sources of information for the government, the NGOs and the development partners to strategically plan their intervention and to efficiently and effectively target the most vulnerable rural households with the most important adaptation packages capable of assisting them in building resilience against the climate change. Results from this study will also serve as reference to design and plan development projects as the saying goes, people make the project and the project makes the development. Limitations of this research include using a small sample size, considering only one county which may limit generalisation of these results. Direction for future research is to financially evaluate local farmers’ adaptation strategies and development partners’ adaptation strategies and to determine combinations of adaptation strategies which will be more welfare enhancing for vulnerable rural households.

## Footnotes

1 In Niger, it is obvious that the year 1984 was marked by the meeting of Maradi about land, water and environment activities to reverse the bad effects of climate change (Agora21, 2000; National Adaptation Program of Action 2013).

2 Statement of H.E. Miguel D’Escoto Brockmann, President of the United Nations General Assembly to the Indigenous Peoples’ Global Summit on Climate Change 20–24 April 2009, Anchorage, Alaska.
